# Optimal adaptive allocation using deep reinforcement learning in a dose‐response study

**DOI:** 10.1002/sim.9247

**Published:** 2021-11-07

**Authors:** Kentaro Matsuura, Junya Honda, Imad El Hanafi, Takashi Sozu, Kentaro Sakamaki

**Affiliations:** ^1^ Department of Management Science, Graduate School of Engineering Tokyo University of Science Katsushika‐ku Tokyo Japan; ^2^ HOXO‐M, Inc. Chuo‐ku Tokyo Japan; ^3^ Department of Systems Science, Graduate School of Informatics Kyoto University Sakyo Ward Kyoto Japan; ^4^ Mathematical Statistics Team RIKEN AIP Chuo‐ku Tokyo Japan; ^5^ Online Decision Making Unit RIKEN AIP Chuo‐ku Tokyo Japan; ^6^ Department of Applied Mathematics ENSTA Paris Paris France; ^7^ Department of Information and Computer Technology, Faculty of Engineering Tokyo University of Science Katsushika‐ku Tokyo Japan; ^8^ Center for Data Science Yokohama City University Yokohama Japan

**Keywords:** adaptive design, clinical trial, dose‐finding, dose‐ranging, optimal design, response‐adaptive

## Abstract

Estimation of the dose‐response curve for efficacy and subsequent selection of an appropriate dose in phase II trials are important processes in drug development. Various methods have been investigated to estimate dose‐response curves. Generally, these methods are used with equal allocation of subjects for simplicity; nevertheless, they may not fully optimize performance metrics because of nonoptimal allocation. Optimal allocation methods, which include adaptive allocation methods, have been proposed to overcome the limitations of equal allocation. However, they rely on asymptotics, and thus sometimes cannot efficiently optimize the performance metric with the sample size in an actual clinical trial. The purpose of this study is to construct an adaptive allocation rule that directly optimizes a performance metric, such as power, accuracy of model selection, accuracy of the estimated target dose, or mean absolute error over the estimated dose‐response curve. We demonstrate that deep reinforcement learning with an appropriately defined state and reward can be used to construct such an adaptive allocation rule. The simulation study shows that the proposed method can successfully improve the performance metric to be optimized when compared with the equal allocation, D‐optimal, and TD‐optimal methods. In particular, when the mean absolute error was set to the metric to be optimized, it is possible to construct a rule that is superior for many metrics.

## 1 INTRODUCTION

Estimation of the dose‐response curve for efficacy and selection of the dose for use in confirmatory phase III trials are one of the most difficult decisions in the drug development process. While too low a dose can result in lack of efficacy, too high a dose can cause unnecessary adverse events.

Various methods have been examined to accurately estimate the dose‐response curve and ensure correct dose selection. Methods for estimating the dose‐response curve include analysis of variance (ANOVA), multiple comparison procedure—modeling (MCP‐Mod) method,^1^ and Bayesian modeling average (BMA)‐based method.^2^, ^3^ These methods are typically used with equal allocation of subjects for simplicity. Various optimal allocation methods, which include adaptive allocation methods, have been studied,^4^ such as the D‐optimal method,^5^ TD‐optimal method,^6^ aMCP‐Mod method,^7^ and Miller's method.^8^ Studies have evaluated some of these methods.^9^, ^10^ One common feature in these studies is the evaluation of the operating characteristics in simulation studies using performance metrics, such as statistical power, accuracy of the estimated target dose, and mean absolute error over the estimated dose‐response curve.

The issue with equal allocation is that the metrics may not be fully optimized due to nonoptimal allocation. Several optimal allocation methods have been proposed in previous studies to overcome this issue, but they rely on asymptotics, and thus sometimes cannot efficiently optimize the performance metric with the sample size in an actual clinical trial. For example, the D‐optimal method minimizes the asymptotic variance of the estimates of the dose‐response model parameters,^5^ and the TD‐optimal method minimizes the asymptotic variance of the estimated target dose.^6^

The purpose of this study is to construct an adaptive allocation rule that can directly optimize the performance metric to be optimized. To achieve this, we use deep reinforcement learning^11^, ^12^ based on the mean and standard deviation of the response for each dose and the number of subjects allocated to each dose. A simulation study was conducted to compare the operating characteristics of the equal allocation (commonly used in actual clinical trials), D‐optimal method, TD‐optimal method, and proposed method.

In Section 2, the performance metrics and the proposed method are described. In Section 3, the simulation settings and results are presented. In Section 4, we summarize and discuss our findings.

## 2 OPTIMAL ADAPTIVE ALLOCATION USING REINFORCEMENT LEARNING

### 2.1 Settings

In most actual dose‐response studies, the doses are limited to predetermined discrete values, and thus, we assume this in this article. The number of doses is denoted by *K*, and the indices of the doses are k=1,…,K, indexed from the lowest dose to the highest dose. The amount of dose is denoted by $d_{k}$, where k=1 is the placebo group with $d_{1}=0$. The total number *N* of subjects to be allocated in a clinical trial is assumed to be predetermined. Each subject is allocated to a dose k∈{1,…,K} and response *Y* is measured. We assume that the clinical team has a performance metric to be optimized, as described in Section 2.2, and determines a method to detect dose‐response and to estimate the dose‐response curve at the end of the trial (eg, ANOVA, MCP‐Mod, or BMA).

In the proposed method, a clinical trial is conducted according to the following steps.

1. At the beginning of the trial, $N_{\text{ini}}$ subjects are allocated equally to k=1,…,K and their responses are obtained.
2. Based on the information obtained so far, each of $N_{\text{block}}$ subjects is probabilistically allocated to one of the *K* doses according to the adaptive allocation rule $\pi^{*}$. Then, their responses are obtained. This step is repeated for b=1,…,B where $B=(N-N_{\text{ini}})/N_{\text{block}}$.
3. At the end of the trial, the dose‐response curve and target dose are estimated, and all performance metrics are evaluated.

In Step 2, the rule $\pi^{*}$ selects a dose *k* so that it can optimize the selected performance metric. The rule $\pi^{*}$ is determined before the start of the trial. In Section 2.3, we explain how the rule $\pi^{*}$ is obtained using deep reinforcement learning.

### 2.2 Performance metrics

When selecting the target dose to be used in a phase III trial, safety and efficacy of the drug are taken into consideration. For the purpose of simulation studies, we simplify the problem and consider only efficacy for dose selection. In simulation studies, the existence of true dose‐response curves is usually assumed to evaluate the methods.^9^, ^10^ The values of the true and estimated dose‐response curves at $d_{k}$ are denoted by $\mu (d_{k})$ and $\hat{\mu }(d_{k})$, respectively. To evaluate the operating characteristics of the methods, the following performance metrics are used in general.^9^, ^10^

#### 2.2.1 Detecting dose‐response

The methods in the previous studies and the proposed method include a decision rule to determine whether the data provides sufficient evidence of dose‐response activity. The probability of identifying the presence of dose‐response is estimated as the percentage of simulated trials in which the decision rule concluded for dose‐response activity. Under a flat dose‐response scenario, it gives the type I error rate, and under a nonflat dose‐response scenario, it provides the power to make the correct identification of dose‐response.

#### 2.2.2 Accuracy of model selection

In several dose‐response curve estimation methods, model selection is done from candidate dose‐response models such as linear, Emax, and sigmoid Emax models. For the accuracy of the model selection, we calculate the percentage of simulated trials in which the dose‐response curve selected in model selection is correct,^13^ and call this metric “MS”. Selecting the correct model is important for estimating the dose‐response curve and target dose with small errors.

#### 2.2.3 Accuracy of a target dose

In this article, the target dose $d_{\text{targ}}$ is defined as the smallest dose that produces an effect difference from placebo greater than or equal to the clinically relevant target effect δ (minimum effective dose, MED). Here, $d_{\text{targ}}$ is a continuous value and is obtained by

$$\begin{array}{ll} d_{\text{targ}}=\underset{d\in [d_{1},d_{K}]}{\text{arg min}}\{\mu (d)\geq \mu (d_{1})+\delta \}. & \end{array}$$

It should be noted that $d_{\text{targ}}$ varies with the true dose‐response curve. We also consider target effect intervals $I_{\text{targ}}^{e}(\eta )=\delta (1\pm \eta )$ (ie, within ±100η% of the target effect) and their corresponding target dose intervals $I_{\text{targ}}^{d}(\eta )$.^10^ The estimated target dose ${\hat{d}}_{\text{targ}}$ is also a continuous value and is defined using the estimated dose‐response curve $\hat{\mu }(d)$ by

$$\begin{array}{ll} {\hat{d}}_{\text{targ}}=\underset{d\in [d_{1},d_{K}]}{\text{arg min}}\{\hat{\mu }(d)\geq \hat{\mu }(d_{1})+\delta \}. & \end{array}$$

We define the accuracy of the estimated target dose by calculating the percentage of simulated trials in which ${\hat{d}}_{\text{targ}}$ is correctly within the interval $I_{\text{targ}}^{d}(0.1)$, and call this metric “TD”. In this study, we evaluate “TD” without rounding ${\hat{d}}_{\text{targ}}$ to the nearest integer because we consider that a better “TD” for the continuous dose also leads to a better “TD” for the discrete dose.

#### 2.2.4 Error in a dose‐response curve

Accurate estimation of the dose‐response curve is relevant not only for estimating target doses, but also for appropriate labeling after approval. To evaluate the accuracy of the dose‐response curve estimation, we calculate the mean absolute error (MAE) between the estimated and true dose‐response curves. In actual clinical trials, it is important to determine the effect compared with the placebo group. Therefore, we calculate the MAE after shifting the dose‐response curve so that the effect in the placebo group is zero.^10^

$$\begin{array}{ll} \text{MAE}=\frac{1}{K-1}\overset{K}{\underset{k=2}{\sum }}\left|\left(\hat{\mu }(d_{k})-\hat{\mu }(d_{1}))-(\mu (d_{k})-\mu (d_{1})\right)\right|. & \end{array}$$

### 2.3 Deep reinforcement learning

In this section, we describe how to use reinforcement learning^11^ to obtain an allocation rule that optimizes the selected metric. To conduct reinforcement learning, the distributions for the dose‐response curve and observation noise must be given to simulate trials. In each simulated trial, a dose‐response curve and responses are probabilistically generated from the distributions. The distributions should reflect the prior beliefs of the clinical team. For example, we can use the candidate models of MCP‐Mod with prespecified probabilities when using it to estimate a dose‐response curve. Similarly, we can use the prior distributions of BMA when using BMA.

In reinforcement learning, a task is formulated as a Markov decision process (MDP), and an important factor is how to specify the state and reward in the MDP. In the application of an MDP, state *s* corresponds to a variable that succinctly describes the information available up to that time point. Now, we consider the situation in which the responses of the bth block have been obtained in Step 2 in Section 2.1. In the proposed method, we define *s* by

$$\begin{array}{ll} s=\left\{{\bar{Y}}_{2}-{\bar{Y}}_{1},{\bar{Y}}_{3}-{\bar{Y}}_{1},\ldots ,{\bar{Y}}_{K}-{\bar{Y}}_{1},{\hat{\sigma }}_{1},\ldots ,{\hat{\sigma }}_{K},\frac{n_{1}}{N},\ldots ,\frac{n_{K}}{N}\right\}, & \end{array}$$

where ${\bar{Y}}_{k}$ and ${\hat{\sigma }}_{k}$ are the mean and standard deviation of the responses of the subjects allocated to dose *k*. The number of subjects allocated to dose *k* up to that time point is denoted by $n_{k}$. Therefore, $\sum_{k=1}^{K}n_{k}=N$ is satisfied at the end of the clinical trial. *s* is a vector of the difference from placebo, the standard deviation, and the proportion of the number of subjects allocated.

We define action *k* to be selected from {1,…,K}. Unlike when we apply the obtained allocation rule, action *k* represents that all $N_{\text{block}}$ subjects within the *b*th block receive the same dose *k* in the learning. This is to speed up and stabilize reinforcement learning.

Next, we define the reward. For each metric selected from those in Section 2.2, we transformed the value into approximately within the range [0,1] at the end of the trial to use the default value of the learning rate hyperparameter in the software. We write $r_{x}$ as the reward when the performance metric is *x*. We define $r_{\text{power}}$, $r_{\text{MS}}$, $r_{\text{TD}}$, and $r_{\text{MAE}}$ as follows:

$$\begin{array}{ll} r_{\text{power}} & =\left\{\begin{array}{cc} 1,\; & \text{if dose‐response is detected under a nonflat model}\\ 0,\; & \text{otherwise} \end{array}\right.\;\\ r_{\text{MS}} & =\left\{\begin{array}{cc} 1,\; & \text{the selected model coincides the true model}\\ 0,\; & \text{otherwise} \end{array}\right.\;\\ r_{\text{TD}} & =\left\{\begin{array}{cc} 1,\; & {\hat{d}}_{\text{targ}}\;\text{is within the interval}\;I_{\text{targ}}^{d}(0.1)\\ 0,\; & \text{otherwise} \end{array}\right.\;\\ r_{\text{MAE}} & =1-2\times \text{MAE}.\; \end{array}$$

We define $Q_{\pi }(s,k)$ as the expected cumulative reward from state *s* by allocating the next block to dose *k* and after that following the allocation rule π (see Appendix for the formal definition). The aim of reinforcement learning is to learn the optimal allocation rule $\pi^{*}$ such that $\max_{k}Q_{\pi }(s,k)$ is maximized for each *s*. When the number of possible values of *s* is finite and small, it is possible to use the backward induction method;^14^ however, this method is not feasible in this case. Instead, we express π using a deep neural network (DNN) and obtain $\pi^{*}$ numerically by reinforcement learning. Several methods have been proposed to learn $\pi^{*}$.^15^, ^16^ Here, we use the proximal policy optimization (PPO) method, a type of deep reinforcement learning, owing to its ease of implementation and high performance.^12^

In the PPO method, the probability π(k|s) of taking action (in our case, dose) *k* under state *s* is represented by a DNN. A DNN with an activation function *f* and consisting of two intermediate layers with *J* units can be described as follows:

$$\begin{array}{llllll} z_{j}^{\left(1\right)} & =f\left(\alpha_{j}^{\left(1\right)}+\underset{i}{\sum }\beta_{ji}^{\left(1\right)}s_{i}\right),\; & z_{j}^{\left(2\right)} & =f\left(\alpha_{j}^{\left(2\right)}+\overset{J}{\underset{j^{\prime }=1}{\sum }}\beta_{jj^{\prime }}^{\left(2\right)}z_{j^{\prime }}^{\left(1\right)}\right),\; & & \;\\ u_{k} & =\alpha_{k}^{\left(3\right)}+\overset{J}{\underset{j^{\prime }=1}{\sum }}\beta_{kj^{\prime }}^{\left(3\right)}z_{j^{\prime }}^{\left(2\right)},\; & \pi (k) & =\text{softmax}(u_{k})=\frac{\exp(u_{k})}{\sum_{k^{\prime }=1}^{K}\exp(u_{k^{\prime }})},\; & & \; \end{array}$$

where $s_{i}$ is an element of *s*, and $\alpha^{\left(1\right)}$, $\beta^{\left(1\right)}$, $\alpha^{\left(2\right)}$, $\beta^{\left(2\right)}$, $\alpha^{\left(3\right)}$, $\beta^{\left(3\right)}$ are the parameters of the DNN.

We estimate $\pi^{*}$ using reinforcement learning. Specifically, we first initialize the parameters of the DNN appropriately to initialize π. Then, we simulate a clinical trial according to the current rule π, and obtain the data of the states and rewards. From these data, the parameters of the DNN are updated based on the gradient to increase the reward. We iteratively simulate trials and update them such that π converges to $\pi^{*}$. See Appendix for the overview of the PPO method.

## 3 SIMULATION STUDY

We conducted a simulation study in a slightly modified setting used by Bornkamp et al^9^ and Dragalin et al.^10^ We compared the performance of the equal allocation, D‐optimal method, TD‐optimal method, and proposed method.

### 3.1 Design of Simulation Study

We assumed a phase II dose‐response study using the MCP‐Mod method, which has been used frequently in actual trials in recent years. Note that it is also possible to use reinforcement learning to directly estimate the dose‐response curve without using MCP‐Mod. Nonetheless, we unified the procedure to use MCP‐Mod for a fair comparison with existing methods and to purely evaluate the efficiency of the allocation rules.

In this trial, five doses (0, 2, 4, 6, and 8 mg) were set, and the total sample size was set to 150 subjects. The clinically relevant target effect was δ=1.3. In MCP‐Mod, candidate dose‐response models (curves) with the values of their shape parameters must be prepared before the start of the trial. The candidates in this trial were Scenarios 1, 4, and 7 in Table 1 with equal probabilities (ie, 1/3 for each), and the maximum effect in the dose range [0,8] was assumed to be 1.65. The response was assumed to be the sum of the dose‐response curve and the observation noise following a normal distribution with mean 0 and variance 4.5.^9^ In MCP‐Mod, multiple testing with a significance level is performed on the candidates at the end of the trial. The models that pass the testing are fitted to the data, and the shape parameters are estimated. Then, model selection is performed using a predetermined criterion. Here, the significance level was set to 0.025, and model selection was performed using Akaike information criterion (AIC). Finally, the performance metrics in Section 2.2 were evaluated using the selected model. Although performance metrics (except power) are not defined under MCP‐Mod in case no model passes the testing, we formally performed model selection using all candidate models and calculated the performance metrics for the evaluation purpose.

**TABLE 1:** Dose‐response scenarios

Scenario no.	Model	Max effect	Formula	$d_{\text{targ}}$	$I_{\text{targ}}^{d}(0.1)$
1	linear	1.65	μ(d)=(1.65/8)d	6.30	(5.67, 6.93)
2	linear	1.65×0.8	μ(d)=(1.32/8)d	7.88	(7.09, 8.00)
3	linear	1.65×1.2	μ(d)=(1.98/8)d	5.25	(4.73, 5.78)
4	Emax	1.65	μ(d)=1.81d/(0.79+d)	2.00	(1.44, 2.95)
5	Emax	1.65×0.8	μ(d)=1.45d/(0.79+d)	6.83	(3.30, 8.00)
6	Emax	1.65×1.2	μ(d)=2.18d/(0.79+d)	1.17	(0.92, 1.52)
7	sigEmax	1.65	$\mu (d)=1.70d^{5}/(4^{5}+d^{5})$	5.06	(4.68, 5.58)
8	sigEmax	1.65×0.8	$\mu (d)=1.36d^{5}/(4^{5}+d^{5})$	7.37	(5.75, 8.00)
9	sigEmax	1.65×1.2	$\mu (d)=2.04d^{5}/(4^{5}+d^{5})$	4.47	(4.24, 4.74)
10	quadratic	1.65	$\mu (d)=(1.65/3)d-(1.65/36)d^{2}$	3.24	(2.76, 3.81)
11	quadratic	1.65×0.8	$\mu (d)=(1.32/3)d-(1.32/36)d^{2}$	5.26	(3.98, 8.00)
12	quadratic	1.65×1.2	$\mu (d)=(1.98/3)d-(1.98/36)d^{2}$	2.48	(2.16, 2.84)
13	exponential	1.65	μ(d)=0.00055(exp(d)−1)	7.76	(7.66, 7.86)
14	exponential	1.65×0.8	μ(d)=0.00044(exp(d)−1)	7.98	(7.88, 8.00)
15	exponential	1.65×1.2	μ(d)=0.00066(exp(d)−1)	7.58	(7.47, 7.67)
16	flat	0	μ(d)=0	‐	‐

*Note:* If the upper of $I_{\text{targ}}^{d}(0.1)$ did not exist or was greater than 8 (maximum dose), the upper was set to 8

**FIGURE 1:**
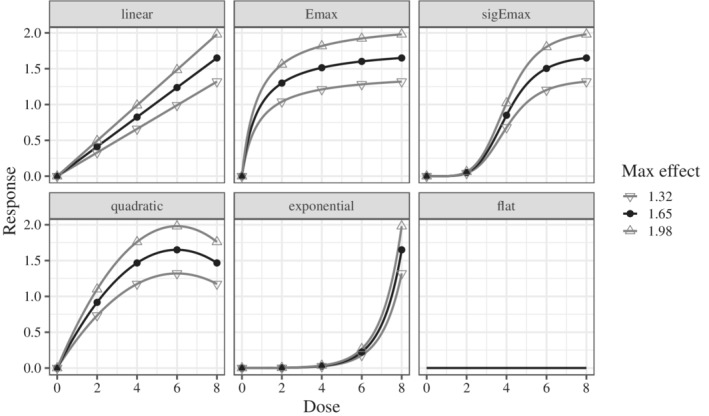
Dose‐response scenarios

For each of the 16 scenarios in Table 1, 10 000 simulated trials were used to estimate the mean of the performance metrics. Scenarios 2, 3, 5, 6, 8, and 9 represent the scenarios where the effect was smaller or larger than the candidates, and Scenarios 10 to 15 represent the scenarios where the model was not included in the candidates. These scenarios were set up to verify the robustness of the allocation rule obtained by the proposed method. Scenario 16 was used to evaluate the type I error rate. These scenarios are illustrated in Figure 1.

### 3.2 Allocation Rule

We used the following eight allocation rules: Equal, D‐optimal 1, D‐optimal 2, TD‐optimal 1, TD‐optimal 2, RL‐power, RL‐MS, RL‐TD, and RL‐MAE. We used the sans‐serif font for rule names to distinguish the objective used in RL, which represents reinforcement learning, from the evaluated performance metrics. The details of the eight allocation rules are described below.

#### 3.2.1 Equal

At the beginning of the trial, 150 subjects were equally allocated to five doses ($n_{1}=n_{2}=n_{3}=n_{4}=n_{5}=30$). This rule is easy to understand and is most frequently used in actual clinical trials.

#### 3.2.2 D‐optimal 1

At the beginning of the trial, the allocation ratios were calculated based on the D‐optimal method^5^ to minimize

(1) $$\begin{array}{ll} -\;\underset{m}{\sum }\frac{p_{m}}{k_{m}}\log\left(\det M_{m}\right), & \end{array}$$

where *m* is the index of each candidate model, $p_{m}$ is the prior probability of model *m* (here, 1/3 for each *m*), $k_{m}$ is the number of parameters of model *m*, and $M_{m}$ is the Fisher information matrix under model *m*. The calculated allocation ratios for each group were 0.30, 0.20, 0.12, 0.09, and 0.29, respectively. The calculated ratios were rounded to integer values using the method by Pukelsheim and Rieder,^17^ and $n_{1}=44$, $n_{2}=30$, $n_{3}=18$, $n_{4}=14$, $n_{5}=44$ were allocated.

#### 3.2.3 D‐optimal 2

The subjects were adaptively allocated based on the D‐optimal method.^5^ More specifically, at the beginning of the trial, 50 subjects were equally allocated to the five doses. Then, after obtaining their responses, we determined the allocation ratios that minimized Equation (1), given the number of allocated subjects and the number of subjects in the next block (ie, by using the options “nold” and “n” in DoseFinding::optDesign function of R). Here, 10 subjects were allocated in the next block. The model probabilities $p_{m}$ (m=1,2,3) were set to 1/3 before the trial, and were updated according to Section 5 in Miller et al^8^ for each block. The shape parameters were not updated and were fixed to those of the candidates (ie, Scenarios 1, 4, and 7). The calculated ratios were rounded to integer values using the method by Pukelsheim and Rieder.^17^ Then, the responses of the 10 allocated subjects were obtained, and the allocation ratios were calculated again to allocate the next 10 subjects. This was repeated until the total number of subjects reached 150.

#### 3.2.4 TD‐optimal 1

At the beginning of the trial, the allocation ratios were calculated based on the TD‐optimal method^6^ to minimize

(2) $$\begin{array}{ll} \underset{m}{\sum }p_{m}\log\left(v_{m}\right), & \end{array}$$

where *m* is the index of each candidate model, $p_{m}$ is the probability of model *m* (here, 1/3 for each *m*), and $v_{m}$ is proportional to the asymptotic variance of the estimated target dose under model *m*. The calculated allocation ratios were 0.31, 0.26, 0.12, 0.18, and 0.14, respectively. According to these ratios, $n_{1}=46$, $n_{2}=39$, $n_{3}=17$, $n_{4}=27$, and $n_{5}=21$ were allocated.

#### 3.2.5 TD‐optimal 2

The subjects were adaptively allocated based on the TD‐optimal method. The procedure was the same as that used in D‐optimal 2, except that the objective function was Equation (2) instead of Equation (1).

#### 3.2.6 RL‐power, RL‐MS, RL‐TD, and RL‐MAE

Because the procedures for constructing these rules are similar, RL‐MAE is explained as an example.

We simulated clinical trials in reinforcement learning using the settings in Sections 2 and 3.1, and learned the allocation rule. In each simulated trial, the dose‐response curve was determined uniformly at random from the scenarios considered in MCP‐Mod (ie, Scenarios 1, 4, and 7), and the observation noise was generated from a normal distribution with mean 0 and variance 4.5. We used $N_{\text{ini}}=50$ and $N_{\text{block}}=10$. In addition, we used ReLU (f(x)=max(0,x)) as the activation function and a DNN consisting of two intermediate layers with 256 units. The settings of the DNN were the default values of the software.^18^ After each simulated trial, the MAE was evaluated. After each 1000 simulated trials, allocation rule π was updated using the accumulated data of the states and MAEs. With 1 000 000 simulated trials in reinforcement learning, the allocation rule $\pi^{*}(k|s)$ was obtained. See Appendix for details on the hyperparameters of the PPO method.

At the beginning of the trial, 50 subjects were allocated equally to the five doses. Thereafter, each time the responses were obtained, each of the 10 subjects was probabilistically allocated to one of the five doses according to the discrete distribution $\pi^{*}(k|s)$. This was repeated until the total number of subjects reached 150.

RL‐power, RL‐MS, and RL‐TD, were the same as RL‐MAE, except that the metrics to be optimized were power, MS, and TD in Section 2.2.

In general, it is known that using *p*‐values without considering adaptive allocation may inflate the type I error rate, and a simulation‐based method to control the type I error rate has been discussed previously.^19^, ^20^ Here, we first calculated the *p*‐values for the flat scenario, and then adjusted the significance level threshold based on the distribution of the *p*‐values. Then, using the adjusted significance level, we simulated the other scenarios and evaluated the performance metrics.

For deep reinforcement learning, we used the RLlib library in Python^18^ and for the MCP‐Mod, D‐optimal, and TD‐optimal methods, we used the DoseFinding package in R.^21^ The code with hyperparameters is available in Supplementary Material, which can be modified according to the requirement.

### 3.3 Results

In this section, the means of the performance metrics obtained from 10 000 simulations for each allocation rule are presented.

**FIGURE 2:**
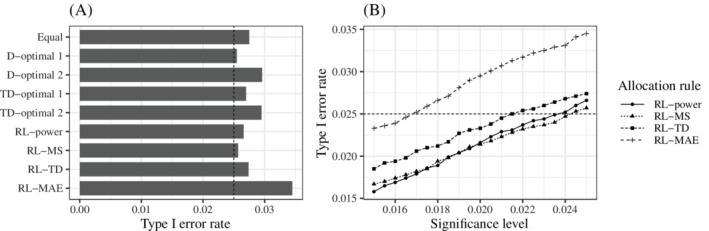
The results for the type I error rate before adjustment

The results for the type I error rate are shown in Figure 2. Figure 2A shows the type I error rate of each rule when the significance level was 0.025. Note that this significance level was based on MCP‐Mod, and the type I error rate was not theoretically guaranteed for adaptive allocation rules. Figure 2B shows the type I error rates of the proposed methods using various significance levels. From these results, we adjusted the significance level to 0.0235 for RL‐power, 0.024 for RL‐MS, 0.021 for RL‐TD, and 0.0165 for RL‐MAE to control the type I error rate. We continued to use a significance level of 0.025 for the other rules, assuming that fluctuations around the 2.5% level were consistent with the Monte Carlo error and the type I error rates were under control. Using these adjusted significance levels, we evaluated the performance metrics for the other scenarios.

The results of the performance metrics (ie, power, MS, TD, and MAE) were similar for the four models (linear, Emax, sigEmax, and quadratic), whereas the results were different for the exponential model. Here, the average results over the all models are shown. For the results of each model, see Figures 1 to 4 in Supplementary Material.

**FIGURE 3:**
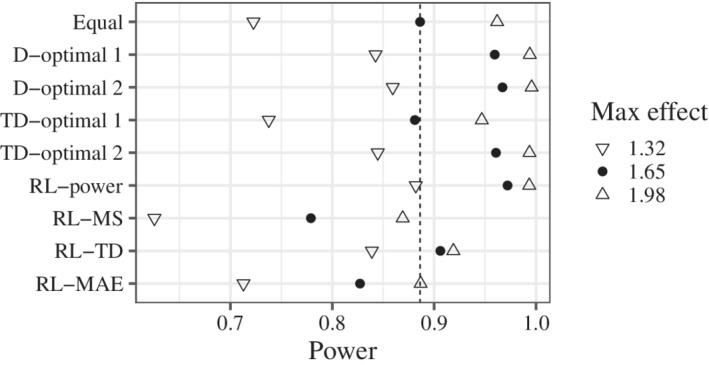
The results for power. The vertical dotted line represents the value of the equal allocation

The results for power are shown in Figure 3. RL‐power certainly improved power. In contrast, RL‐MS and RL‐MAE worsened the power. The lower average power of RL‐MAE may be due to the much lower power when the exponential model was true (see Supplementary Material). Notably, RL‐power had high power even when the true maximum effect was smaller and larger than the candidates, even though RL‐power was trained assuming a maximum effect of 1.65.

**FIGURE 4:**
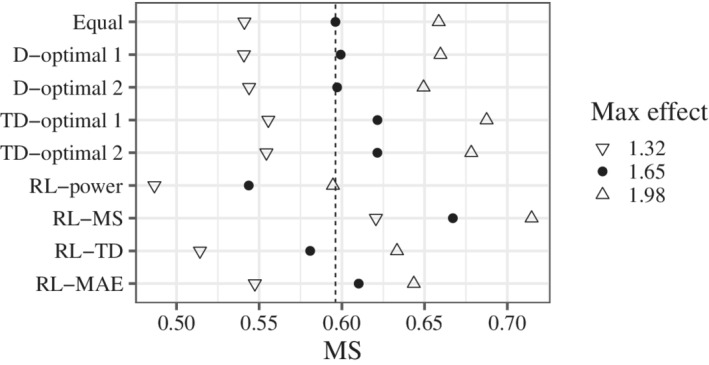
Probability of selecting the true model

**FIGURE 5:**
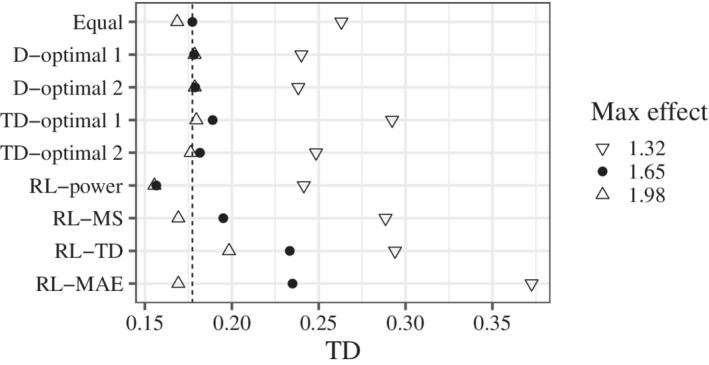
Probability that the estimated target dose is within the interval $I_{\text{targ}}^{d}(0.1)$

**FIGURE 6:**
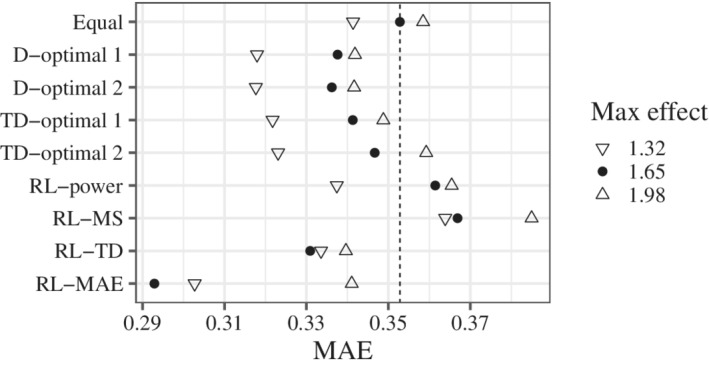
The results for MAE. Smaller MAE implies better accuracys

The results for MS, TD, and MAE (Figures 4‐6) were calculated from simulations where multiple testing was significant. We confirmed that the results were almost the same, even if we included the simulations in which the testing was not significant. The results for the MS are shown in Figure 4. RL‐MS certainly improved the MS. In contrast, RL‐power worsened the MS. RL‐MS was also effective in scenarios different from the candidates. The results for the TD are shown in Figure 5. Better results were obtained when the maximum effect was smaller than that of the candidates. This may be because of the wider range of $I_{\text{targ}}^{d}(0.1)$. RL‐TD and RL‐MAE improved the TD. Note that these rules were better than TD‐optimal 1 and 2. In contrast, RL‐power worsened the TD. The results for the MAE are shown in Figure 6. RL‐MAE improved the MAE. In contrast, RL‐MS worsened the MAE. RL‐MAE was also effective in scenarios that were different from the candidates.

In summary, these results showed that the proposed method improves not only the performance metric used for optimization, but also many other metrics. In particular, RL‐MAE was superior in most metrics for correctly estimating the dose‐response relationship for phase III trials.

**FIGURE 7:**
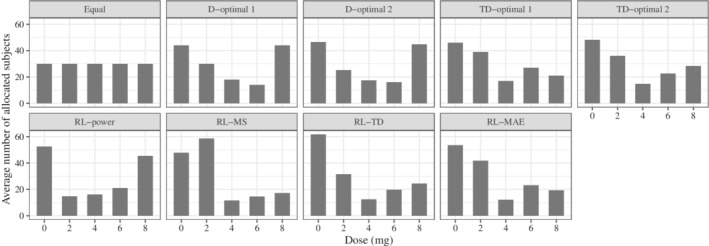
The results for the average number of subjects allocated

The average number of subjects allocated to each dose is shown in Figure 7. This figure shows that the proposed methods tended to allocate more subjects to 0 mg than Equal. In addition, RL‐MS and RL‐MAE tended to allocate more subjects to 2 mg. Since the allocation that optimizes power for the contrast test (assuming the same variance across the dose groups) should be the allocation that places half of the subjects on placebo and the other half on the dose providing the maximum effect, it is natural that RL‐power tended to allocate more subjects to 0 and 8 mg. Since 0, 2, and 8 mg are likely to be important in distinguishing the flat and Emax models from the rest, it is natural that more subjects will be allocated to these doses. For the results of each model, see Figures 5 to 7 in Supplementary Material.

Note that the good performance of RL‐MAE was not only due to the nonuniform allocation, but also from the adaptivity of the allocation. In fact, we confirmed that the performance does not improve if we use a fixed design with the number of subjects equal to the average of those of RL‐MAE in Figure 7. See Supplementary Material for details.

## 4 DISCUSSION

We showed that deep reinforcement learning with an appropriately defined state and reward can be used to construct adaptive allocation rules that can directly optimize the performance metrics to be optimized. In general, reinforcement learning becomes difficult when the reward (ie, the performance metric evaluated at the end of each trial) is delayed, and the observation is noisy. Phase II trials have these difficulties, and it is not obvious whether reinforcement learning works successfully to address the same. Nonetheless, we have shown that it can work well if we appropriately design and choose the Markov decision process as well as the learning algorithm and hyperparameters.

A limitation of this method is that it is difficult to visualize and understand the obtained allocation rule intuitively because the state is multidimensional. An allocation example of RL‐MAE in a single simulated trial is shown in Figure 8 in Supplementary Material. Interactive software such as a Shiny application may help team members to understand the rule. In a clinical trial protocol, it is necessary to specify the assumptions (state, action, and reward) and the selected performance metric, and it would be helpful to show allocation examples.

In the definition of state, we used the differences from the placebo (eg, ${\bar{Y}}_{2}-{\bar{Y}}_{1}$) to avoid making assumptions about the placebo response. When we have a specific prior distribution reflecting the background knowledge on the placebo response, it is also natural to define the state by

$$\begin{array}{ll} s=\left\{{\bar{Y}}_{1},{\bar{Y}}_{2},\ldots ,{\bar{Y}}_{K},{\hat{\sigma }}_{1},\ldots ,{\hat{\sigma }}_{K},\frac{n_{1}}{N},\ldots ,\frac{n_{K}}{N}\right\}. & \end{array}$$

We also simulated the proposed methods with slightly modified states, rewards, and model probabilities, which retrieved in general similar results. Nonetheless, it may be possible to slightly improve the performance by further tuning these settings.

Although the simulation study was conducted assuming Gaussian noise with the MCP‐Mod method, the proposed method can also be applied to other settings (eg, binary response) and other methods (eg, ANOVA and BMA). For example, if the variance of the observation noise is unknown, we will assume the prior distribution of the variance to generate it in reinforcement learning. Because the settings are quite standard in practice, we can expect that the proposed method can cover a wide range of actual clinical trials.

Results showed that the proposed methods was required to adjust the significance level to control the type I error rate. Therefore, developing a statistical test that is theoretically guaranteed under adaptive allocation is an important research topic. The optimization of power (RL‐power) did not necessarily lead to improvements in other performance metrics. On the other hand, RL‐MAE showed good results not only for MAE, but also for other metrics. This seems to intuitively correspond to the fact that if the dose‐response curve itself is estimated with a small error, then the other purposes are achievable. For this reason, it seems natural to use RL‐MAE if the focus is not on any particular metric. Note that it is theoretically the best to allocate all subjects to 0 and 8 mg to maximize the power under the scenarios used in the learning. RL‐power indeed allocated many subjects to 0 and 8 mg, but there still exists a gap from this ideal allocation, which may be due to incomplete learning. Therefore, further tuning of the parameters and neural network may improve the performance. The results described in Supplementary Material showed that RL‐MS and RL‐MAE performed poorly when the exponential model was true. This indicates that if the candidate models considerably differ from the true model, the allocation rules obtained from the learning may not perform well. It may be important to specify the distribution that will generate many possible models in reinforcement learning. In fact, by including the exponential model in the learning, we obtained good performance without sacrificing the performance for the other models (see Supplementary Material).

When the proposed method is used, the number of subjects allocated could be unbalanced. When the imbalance must be taken into consideration for safety or ethical reasons, the number of subjects allocated equally at the beginning of the trial can be increased or a penalty can be incorporated in the reward if the number of subjects at a dose does not reach the threshold. Although we allocated subjects probabilistically according to the discrete distribution $\pi^{*}(k|s)$ when applying the obtained rule, we can also use the rounding method, such as the one reported by Pukelsheim and Rieder.^17^ The results were generally similar.

Although we used one performance metric for optimization, any metric can be used, including a combination of multiple metrics, because the method does not depend on the specific properties of the performance metric. Since many factors other than dose‐response are involved in actual phase III trials, it is also important to develop an appropriate performance metric for the success of phase III trials. Furthermore, we believe that it is possible to extend this approach for the stopping rules for success or futility by adding a stopping option as one of the actions and defining an appropriate reward for the option. It remains to be verified in which situations this will apply.

## Supporting information


**Data S1** Supplementary Material.

## Figures and Tables

**FIGURE A1:**
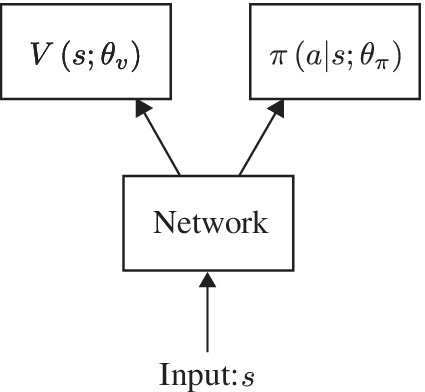
Actor‐Critic PPO network

## Data Availability

Source code of the proposed method is available on https://github.com/MatsuuraKentaro/Optimal_Adaptive_Allocation_in_a_Dose‐Response_Study

## Appendix

In this appendix, we introduce the overview of reinforcement learning techniques used in the proposed method. See, for example, Sutton and Barto^11^ for general introduction to reinforcement learning.

### A.1 Preliminaries for reinforcement learning

Formally, a reinforcement learning problem can be characterized by a Markov Decision Process defined by the 4‐tuple (𝒮,𝒜,P,R):

- State space of the environment 𝒮: At each time step *t* the agent observes a state of the environment denoted $s_{t}\in 𝒮$. The initial state is sampled from an initial distribution on 𝒮.
- Action space 𝒜: At time step *t*, the agent selects an action $a_{t}\in 𝒜$ according to a *policy* π as $a_{t}∼\pi (\cdot |s_{t})$, where π(·|s) is a probability distribution over 𝒜 that represents the strategy of the agent when the state is *s*.
- Transition probability $P(s^{\prime }\;|\;s,a)$: Given the action $a_{t}$ and the state $s_{t}$, the environment evolves into a new state $s_{t+1}$ with probability $P(s_{t+1}\;|\;s_{t},a_{t})$.
- Reward function $R(s,a,s^{\prime })$: The agent receives a reward $r_{t}=R(s_{t},a_{t},s_{t+1})$ when taking action $a_{t}$ at state $s_{t}$ and the new state becomes $s_{t+1}$. We denote by $r(s_{t},a_{t})$ the expected value of $R(s_{t},a_{t},s_{t+1})$ given $\left(s_{t},a_{t}\right)$.

The interaction between the agent and the environment lasts for an episode, that is limited by time or by reaching a terminal state, and then the process restarts. For simplicity of notation we denote by *T* the end of the interaction and it can be finite or infinite.

The return $G_{t}$ defined by

$$\begin{array}{ll} G_{t}=\overset{T}{\underset{k=t}{\sum }}\gamma^{k-t}r(s_{k},a_{k}) & \end{array}$$

is the discounted cumulative reward after time *t* with a discount factor γ∈[0,1]. Small values of γ leads the agent to focus on short‐term rewards while a large value favors long‐term rewards. The goal of the agent is to select at each state the action that will lead the highest expected cumulative discounted reward.

The value function $V_{\pi }$ of a state *s* is the expected return from this state following the policy π and denoted as

$$\begin{array}{ll} V_{\pi }(s)={𝔼}_{\pi }(G_{t}|s_{t}=s), & \end{array}$$

where the expectation is taken over all possible stochastic trajectories under π. The state‐action value function $Q_{\pi }$ is the expected return from state *s* by taking action *a* and after that following the policy π:

$$\begin{array}{ll} Q_{\pi }(s,a)={𝔼}_{\pi }(G_{t}|s_{t}=s,a_{t}=a). & \end{array}$$

The advantage function $A_{\pi }$ is defined by

$$\begin{array}{ll} A_{\pi }(s,a)=Q_{\pi }(s,a)-V_{\pi }(s). & \end{array}$$

The advantage expresses how much better or worse the reward obtained by action *a* in state *s* is compared with the average expected reward $V_{\pi }(s)$ from state *s*.

### A.2 Proximal policy optimization

Many algorithms have been proposed to optimize π. For example, deep Q‐network (DQN) tries to find a good policy indirectly by estimating Q‐values and choosing the action maximizing the estimated Q‐value for each state *s*.^22^ In contrast, policy optimization methods perform a gradient update directly on the parameters of a policy.

Proximal Policy Optimization (PPO) belongs to these methods, which parameterizes the policy as $\pi (a_{t}|s_{t};\theta )$ and update θ directly using the observed rewards $r_{t}$. For update of θ, PPO considers the following objective function to optimize in order to estimate the optimal parameter $\hat{\theta }$.

Let us denote the probability ratio between the old and new policies as

$$\begin{array}{ll} f(\theta ,t)=\frac{\pi_{\theta }(a_{t}|s_{t})}{\pi_{\theta_{\text{old}}}(a_{t}|s_{t})}, & \end{array}$$

where $\theta_{\text{old}}$ is the parameter obtained in the last update. Conceptually, PPO tries to improve the policy by maximizing

(A1)
$$\begin{array}{ll} J(\theta )={𝔼}_{t}(f(\theta ,t){Â}_{\theta_{\text{old}}}(s_{t},a_{t})), & \end{array}$$

where ${𝔼}_{t}$ indicates the empirical average over a finite batch of trajectories (the histories of the states, actions, and rewards through timesteps) and ${Â}_{\theta_{\text{old}}}(s_{t},a_{t})$ is an approximation of the advantage function (this approximation is discussed later).

Still, it is empirically known that maximizing this objective with respect to θ, without a restriction on the distance between $\theta_{\text{old}}$ and θ, results in instability and too aggressive updates. PPO solves this problem by imposing a constraint on f(θ,t) to be within a small interval around 1, precisely [1−ϵ,1+ϵ]. To be more specific, PPO replaces $f(\theta ,t){Â}_{\theta_{\text{old}}}(s_{t},a_{t})$ in Equation (A1) with

$$\begin{array}{ll} \min\{f(\theta ,t){Â}_{\theta_{\text{old}}}(s_{t},a_{t}),\text{clip}(f(\theta ,t),1-\epsilon ,1+\epsilon ){Â}_{\theta_{\text{old}}}(s_{t},a_{t})\}, & \end{array}$$

where clip(x,a,b)=min{max{x,a},b} for a<b.

The estimator of ${Â}_{\theta_{\text{old}}}(s_{t},a_{t})$ is built using generalized advantage estimation (GAE)^23^ given by

$$\begin{array}{ll} {Â}_{\theta_{\text{old}}}(s_{t},a_{t})=\delta_{t}+(\gamma \lambda )\delta_{t+1}+\cdots +{\left(\gamma \lambda \right)}^{T-t+1}\delta_{T-1}, & \end{array}$$

where λ∈[0,1] is a hyperparameter and $\delta_{t}=r_{t}+\gamma V_{\theta_{\text{old}}}(s_{t+1})-V_{\theta_{\text{old}}}(s_{t})$.

Here note that the estimation of the advantage function involves the learned value function $V_{\theta }(s)$. The authors of PPO suggest to use the Actor‐Critic method to estimate the value function. As the name suggest, it has two components: the actor and the critic. The actor corresponds to the policy π and is used to choose the action for the agent. The critic corresponds to the value function *V*. The Actor‐Critic is represented by a shared neural network θ, which then branches into two heads (one for the actor and one for the critic) at the end of the architecture (Figure A1). Therefore θ includes the parameter $\theta_{v}$ for the value function *V* and $\theta_{\pi }$ for the policy π. So that the critic approximates the actual return well, PPO imposes the penalty for the approximation error given by

$$\begin{array}{ll} -\;{\left(V_{\theta }(s_{t})-V_{t}^{\text{target}}\right)}^{2}, & \end{array}$$

where $V_{t}^{\text{target}}=G_{t}$ is the observed return (from the simulation) at time *t*, $V_{\theta }(s_{t})$ is the estimated value function from the neural network at state $s_{t}$.

In summary, PPO maximizes the following objective function:

$$\begin{array}{ll} J{\left(\theta \right)}^{\text{CLIP}} & ={𝔼}_{t}(\min\{f(\theta ,t){Â}_{\theta_{\text{old}}}(s_{t},a_{t}),\text{clip}(f(\theta ,t),1-\epsilon ,1+\epsilon ){Â}_{\theta_{\text{old}}}(s_{t},a_{t})\}-c_{1}{\left(V_{\theta }(s_{t})-V_{t}^{\text{target}}\right)}^{2}+c_{2}S[\pi_{\theta }](s_{t})),\; \end{array}$$

where $c_{1},c_{2}$ and ϵ are hyperparameters. Here *S* in the last term is some entropy function such as $S[\pi ](s)=-\sum_{a\in 𝒜}\pi (a|s)\log\pi (a|s)$, which gives a bonus to a policy that explores a variety of actions.

### A.3 Algorithm

For each update of the neural network parameter θ, an actor collects data of $T_{\text{train}}$ timesteps over multiple episodes. Then we construct the objective function given in the last section on these $T_{\text{train}}$ timesteps data, and maximize it with the stochastic gradient descent (SGD) or Adam algorithm.

Algorithm 1 PPO Algorithm 1

### A.4 Specification in our problem

In our problem setting, the episode, time *t*, and policy π correspond to the simulated trial, block *b*, and allocation rule π, respectively. For the reward, *r* had a value described in Section 2.3 if the time was at the end of the trial, but otherwise r=0. We used the discount factor γ=1 (ie, there is no discount). Therefore, the expected return is equivalent to the expected value of the reward at the end of the trial. For the advantage, we used λ=1. For the objective function, we used $c_{1}=1$, $c_{2}=0$, and ϵ=0.3. For the update of θ, we used E=1000000 and $T_{\text{train}}=10\;000$. That is, θ was updated after each $T_{\text{train}}/B=10\;000/10=1\;000$ simulated trials. To maximize the object function, we used the SGD algorithm with the minibatch size = 200, the stepsize (ie, learning rate) = 0.00005, and the number of epochs = 20. Although we used the default values of the software for λ, $c_{1}$, $c_{2}$, ϵ, and the stepsize, we had to tune $T_{\text{train}}$ and the minibatch size.
