# Supplementary material for: Optimal adaptive allocation using deep reinforcement learning in a dose‐response study
Source: Stat Med. 2021 Nov 7;41(7):1157–71. doi: 10.1002/sim.9247 (PMC9298337; doi:10.1002/sim.9247)
Supplement: Supplementary file 1 — Data S1 Supplementary Material. [file SIM-41-1157-s001.pdf]

Supplementary Materials

The following figures supplement the results of the simulation study.

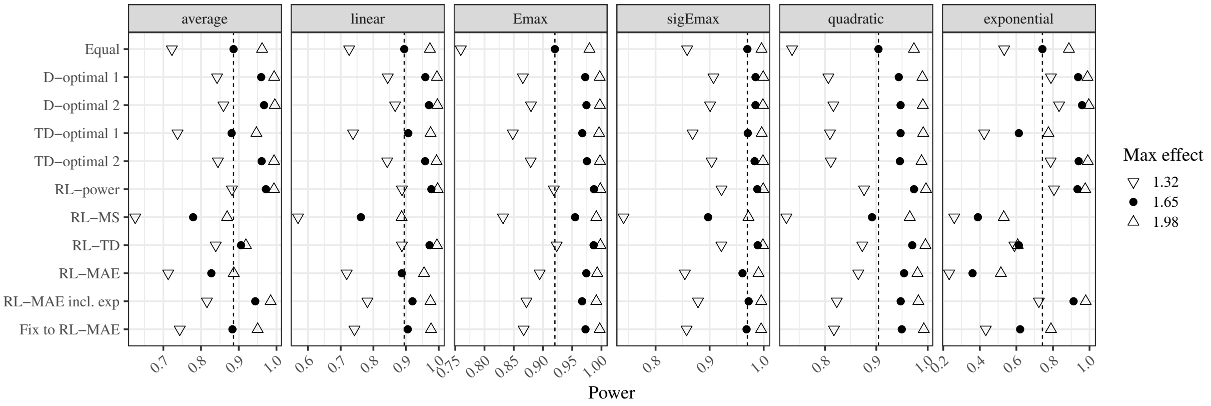

Figure 1: The results for power. RL-MAE incl. exp represents that simulated data in reinforcement learning is generated from the four models (linear, Emax, sigEmax, and exponential) with equal probabilities. Fix to RL-MAE represents a fixed design with the number of subjects equal to the average of those of RL-MAE.

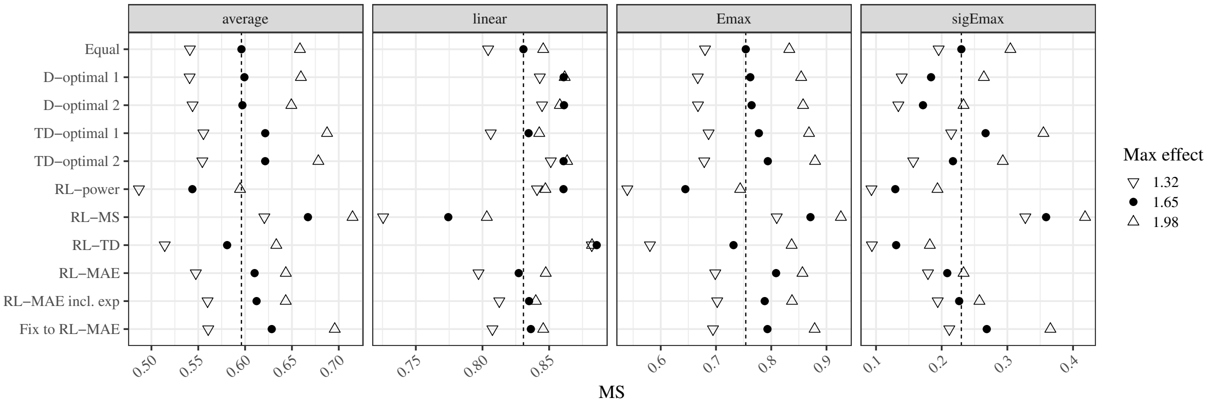

Figure 2: Probability of selecting the true model.

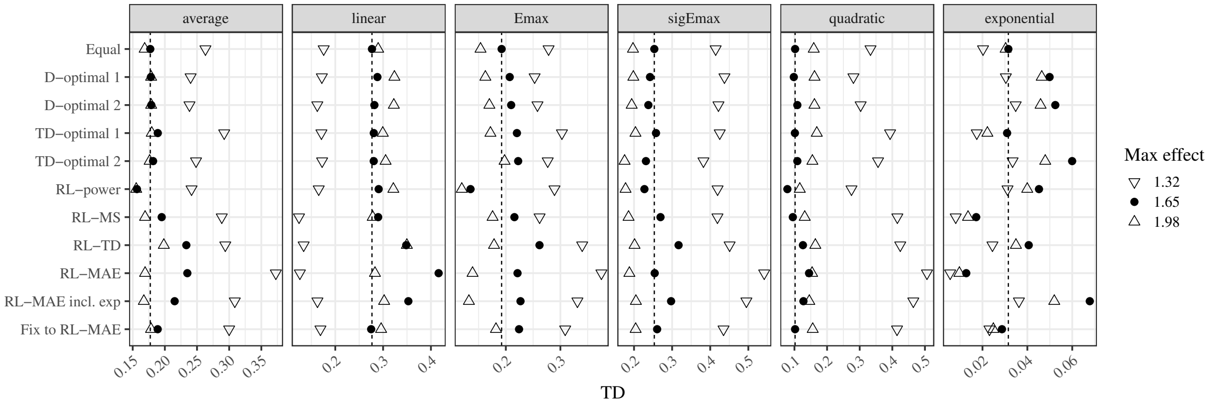

Figure 3: Probability that the estimated target dose is within the interval $I^d_{\mathrm{targ}}(0.1)$.

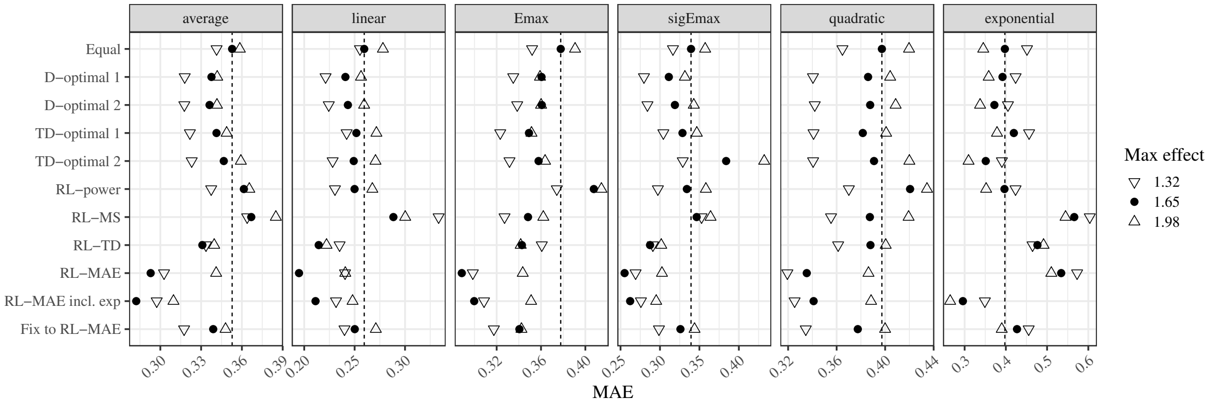

Figure 4: The results for MAE. Smaller MAE implies better accuracy.

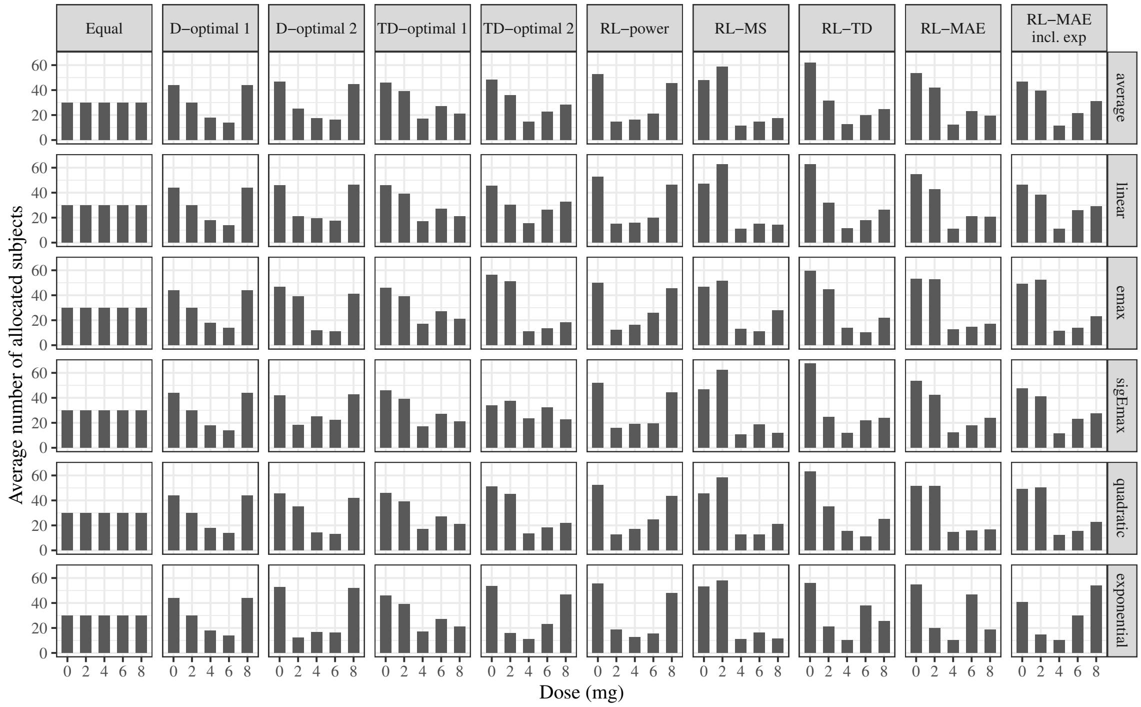

Figure 5: The results for the average number of subjects allocated when the maximum effect was 1.65.

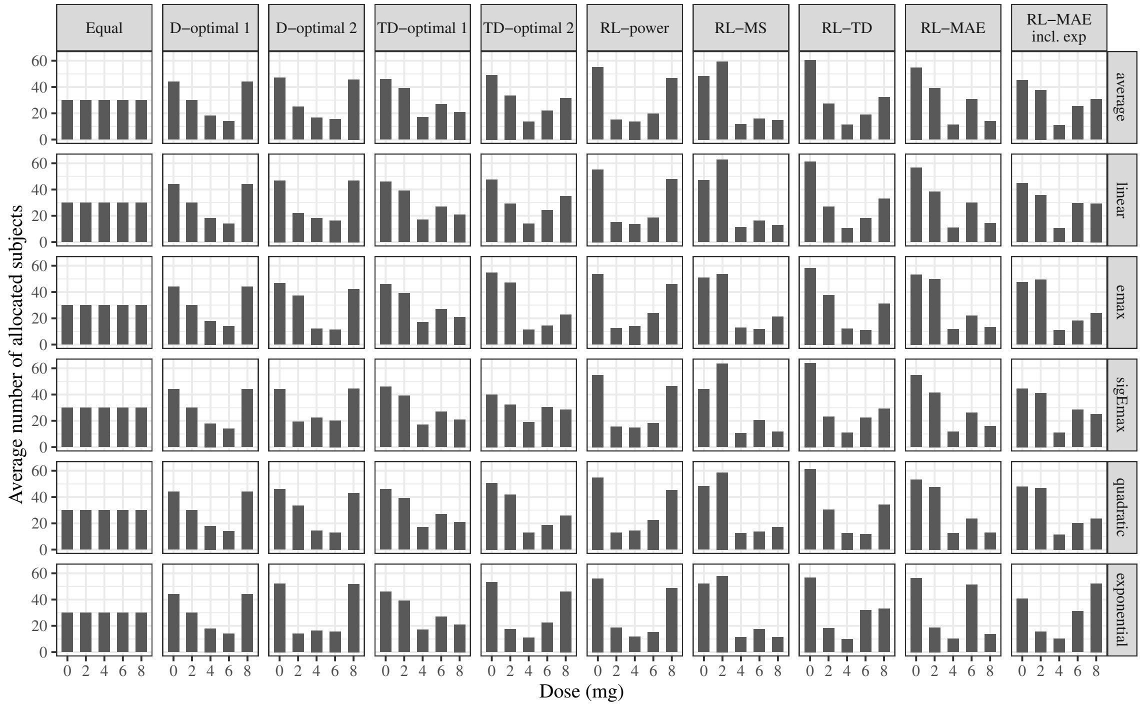

Figure 6: The results for the average number of subjects allocated when the maximum effect was 1.32.

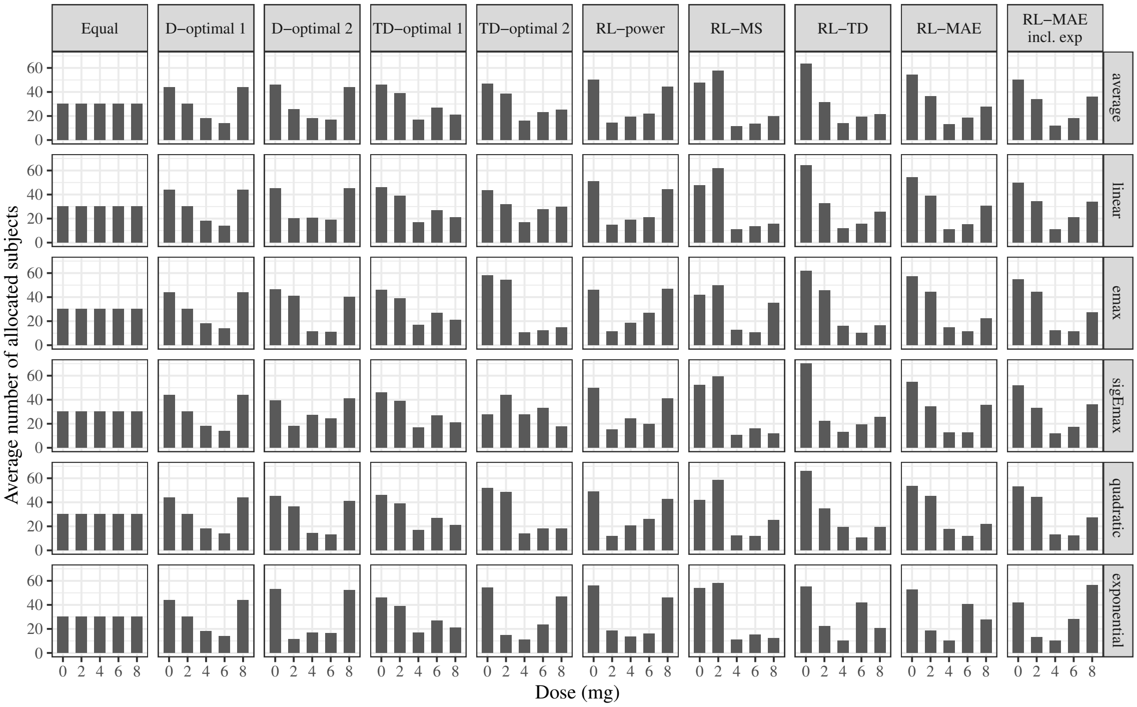

Figure 7: The results for the average number of subjects allocated when the maximum effect was 1.98.

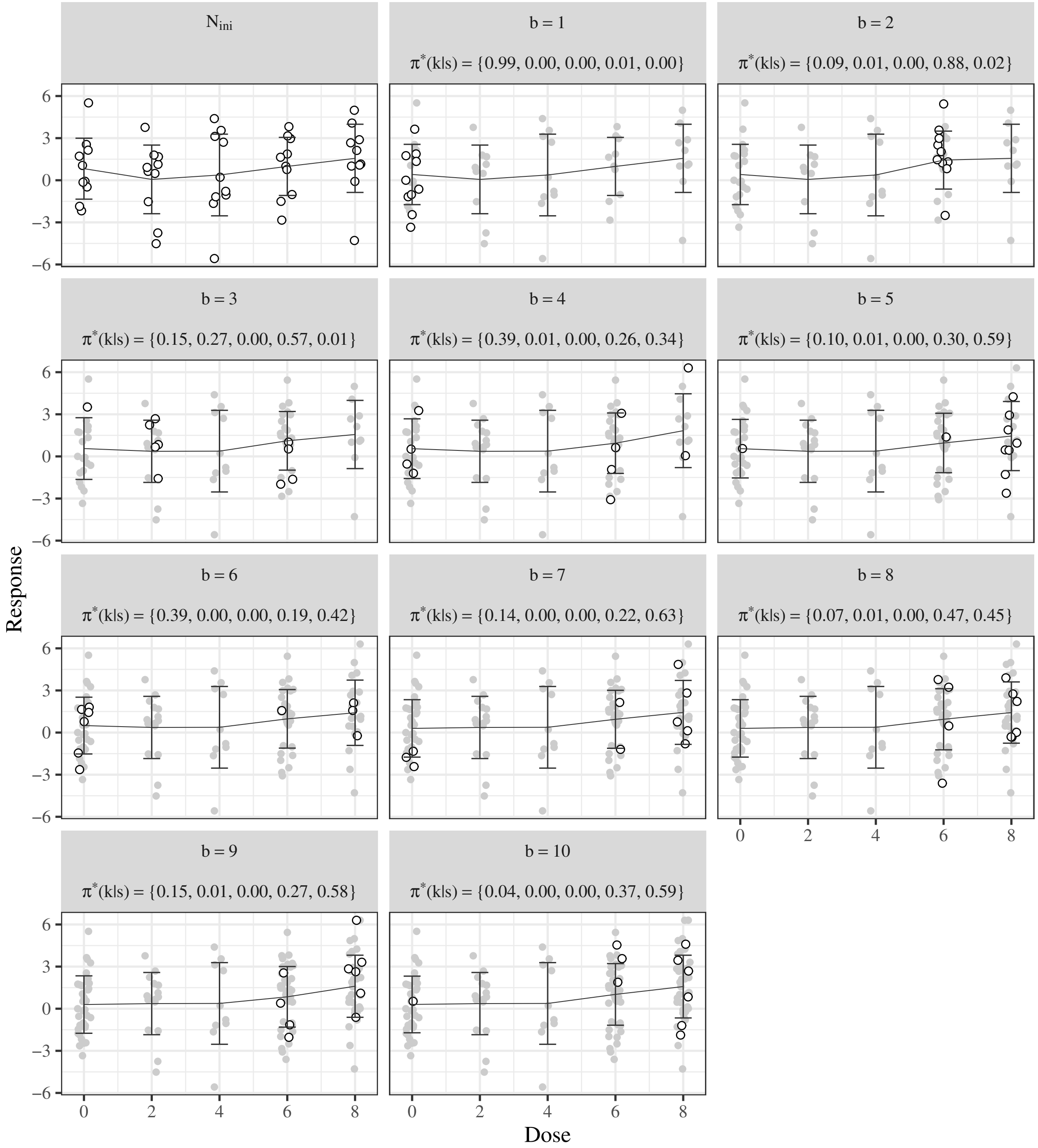

Figure 8: An allocation example of RL-MAE in a simulated trial when the linear model was true and the maximum effect was 1.65. b represents the situation after allocating the subjects in the b-th block and obtaining their responses. The values of $\pi^*(k|s)$ were calculated before allocating the subjects in the b-th block. The open circles represent the responses of the subjects allocated in the b-th block. The gray circles represent the responses of the subjects allocated in the previous blocks. The black lines represent the means of the responses at each dose, and the error bars represent ± 1 SD. At the end of this simulated trial, the MCP-Mod method calculated the p-value as 0.0067 and selected the linear model.
